# IL-1*β* Pretreatment Improves the Efficacy of Mesenchymal Stem Cells on Acute Liver Failure by Enhancing CXCR4 Expression

**DOI:** 10.1155/2020/1498315

**Published:** 2020-07-07

**Authors:** He Nie, Fangmei An, Jie Mei, Cheng Yang, Qiang Zhan, Qinglin Zhang

**Affiliations:** ^1^Department of Gastroenterology, Wuxi People's Hospital Affiliated to Nanjing Medical University, Wuxi, Jiangsu 214023, China; ^2^Department of Oncology, Wuxi People's Hospital Affiliated to Nanjing Medical University, Wuxi 214023, China

## Abstract

**Background:**

Mesenchymal stem cells (MSCs), with the powerful metabolic and functional supporting abilities for inflammatory diseases, may be an effective therapeutic strategy for acute liver failure (ALF). However, the efficacy of MSCs can still be promoted if pretreatment is applied to enhance their poor migration towards the damaged liver. The purpose of this study is to determine the effect of IL-1*β* pretreatment on the efficacy and homing ability of MSCs in ALF.

**Methods:**

MSCs were isolated by the whole bone marrow adherence method and characterized. The efficacy and homing ability of IL-1*β*-pretreated MSCs (Pre-MSCs) were examined in a rat ALF model and compared with that of MSCs and normal saline. Then, Western blot was performed to detect the c-Met and CXCR4 expression of MSCs and Pre-MSCs and followed by flow cytometry to detect the meaningful indicators. Finally, the migration abilities of different cells and different conditions were tested by the Transwell migration assay.

**Results:**

MSCs of ideal purity were successfully isolated and cultured. Comparing with MSCs, Pre-MSCs had significantly better efficacy on improving the survival rate and liver function of ALF rats. Further analyses of damaged liver tissues showed that IL-1*β* pretreatment significantly enhanced the efficacy of MSCs on suppressing liver necrosis. Besides, Pre-MSCs exhibited better effects in inhibiting apoptosis and activating proliferation. The results of tracing experiments with CM-Dil-labeled cells confirmed that more cells migrated to the damaged liver in the Pre-MSC group. In terms of mechanism, the CXCR4 expression was significantly enhanced by IL-1*β* pretreatment, and an increased migration ability towards SDF-1 that could be reversed by AMD3100 was found in Pre-MSCs.

**Conclusion:**

IL-1*β* pretreatment could enhance the homing ability of MSCs at least partially by increasing the expression of CXCR4 and further improve the efficacy of MSCs on ALF.

## 1. Introduction

Acute liver failure (ALF), which is caused by sudden massive hepatocyte death or dysfunction, is a life-threatening clinical syndrome characterized by decompensation of liver function and coagulopathy [1]. Although the efficacy of ALF therapy has been significantly improved with the incessant development of medicine, the mortality rate is still as high as 40% to 62.2% [2, 3]. Liver transplantation is the best option for ALF, but its clinical application is seriously limited by the lack of available organs, immune rejection, and other factors. With its quick function in synthesis, detoxification, and bilirubin excretion, hepatocyte transplantation may possibly overcome some limitations of liver transplantation. However, limited cell sources and poor engraftment of cells hinder the clinical application of this therapeutic approach [4]. Therefore, it is urgent to explore novel strategies for ALF treatment. In recent years, the progress of stem cells in the clinical application provides a new insight into the therapy of ALF.

Mesenchymal stem cell (MSC) is one type of adult stem cells with the characteristics of high proliferative activity, multidirectional differentiation, and low immunogenicity. In addition, MSCs have the capacities of anti-inflammation, antiapoptosis, proliferative promotion, and immune regulation. So MSCs are widely applied in the treatment of multiple diseases including ALF [5, 6]. In an ALF model induced by acetaminophen, intravenous administration of MSCs significantly improved liver function and regeneration as well as antioxidant capacity, thus enhancing the survival rate of ALF animals [7]. Satisfactory efficacy of MSCs was also achieved in other ALF models induced by concanavalin A, D-galactosamine, carbon tetrachloride (CCl_4_), etc. [6, 8]. The powerful metabolic and functional supporting actions of MSCs, by secreting cytokines based on disease microenvironment, are the crucial factors to realize its therapeutic potentials, and the paracrine is the main mechanism [6, 8]. However, most MSCs used intravenously are sequestered in the lungs, and few can migrate and engraft in the damaged tissues, which seriously limited the benefit of MSCs [9]. So, improving the homing ability may enhance the efficacy of MSCs.

The migration ability and efficacy of MSCs can be significantly improved by various pretreatment protocols before application *in vivo* [9, 10]. As confirmed by Wang et al., gene-modified c-Met overexpression significantly improved the homing ability to the damaged liver and the anti-ALF efficacy of MSCs [11]. Deng et al. showed that pretreatment with serum from ALF rats significantly elevated the expression of CXCR4, a crucial regulator for MSC chemotaxis [12]. However, the unguaranteed safety for genetic engineering and the uncontrollable concentration of constituents in liver injury serum seriously limit their clinical application as pretreatment protocols. As one of the major inflammatory factors, IL-1*β* plays an important role in the progression of inflammation including ALF [13]. On the one hand, with the proteolysis of caspase-1, IL-1*β* activates NF-*κ*B signal-based IL-1R-MyD88 pathway and thereby initiates or enhances the proinflammatory response, leading to apoptosis, organ damage, and animal death [13]. On the other hand, IL-1*β* upregulates the expression of Pyrin-only protein 1 (POP1) and POP2, thus forming a feedback loop to prevent excessive inflammation [14]. Also, IL-1*β* plays an important role in the recruitment of inflammatory cells and cancer cell migration [15–17]. It is reported that the adhesion capacity of T cells could be enhanced by IL-1*β* [16]. Meanwhile, IL-1*β* could upregulate the expression of CXCR4 and promote the metastasis of squamous cell carcinoma [17]. However, acting as a cytokine which MSCs will inevitably contact after administration *in vivo*, it is unclear whether IL-1*β* pretreatment could enhance the homing ability and further improve the efficacy of MSCs on ALF.

In this study, the IL-1*β*-pretreated MSCs were administrated to ALF rat models, and the promising efficacy was observed. The impact of IL-1*β* pretreatment on the homing ability of MSCs and the relevant mechanism were also preliminarily studied. To conclude, we found that IL-1*β* pretreatment could enhance the homing ability of MSCs by increasing the expression of CXCR4, thus improving the efficacy of MSCs on ALF.

## 2. Materials and Methods

### 2.1. Animals

Adult male Sprague-Dawley rats (6-8 weeks old, 180-200 g) were purchased from Changzhou Cavens Laboratory Animal Co., Ltd. All rats were housed in a specific pathogen-free environment of constant humidity and temperature with a 12/12 h light/dark cycle, according to the guidelines of Nanjing Medical University. All experimental procedures were approved by the Supervisory Committee of Nanjing Medical University Animal Council.

### 2.2. Preparation and Characterization of MSCs

Rat bone marrow MSCs were isolated and cultured by the whole bone marrow adherence method as described in our previous study [18]. Briefly, the rats were sacrificed by anesthesia, and the bone marrow of femora and tibiae was washed out with phosphate-buffered saline (PBS). The collected bone marrow was dispersed, centrifuged at 1,500 rpm for 5 min in sterile tubes, and resuspended in Dulbecco's modified Eagle's medium (DMEM): Nutrient Mixture F-12 (DMEM/F-12; Life Technologies, Inc., Gaithersburg, MD, USA) culture medium supplemented with 10% (*v*/*v*) FBS (HyClone, UT, USA). The resuspended cells were cultured in an incubator at 37°C with a humidified atmosphere containing 5% (*v*/*v*) carbon dioxide (CO_2_). All nonadherent cells were removed with the change of culture medium performed every 72 h. When the cell confluence degree reached ∼70% in primary cultures, the subculture was carried out. The primary culture cells were labeled P0, the first subcultured cells were labeled P1, and the number of labeling increased successively.

The morphology of MSCs was observed by an inverted microscope (Nikon TE2000U, Tokyo, Japan). The immunophenotypic analyses were carried out at P4 cells by flow cytometry, and the antibodies included anti-rat CD90, C105, CD45, and CD34 (eBioscience, San Diego, CA, USA). For differentiation experiments, the P4 MSCs were incubated in adipogenic or osteogenic medium (Cyagen, Guangzhou, China) for 2 weeks. And then, Oil Red O or Alizarin Red S staining was performed.

For pretreatment, the P4 MSCs were chosen. Briefly, the culture medium containing 20 ng/mL IL-1*β* (PeproTech, Rocky Hill, NJ, USA) was used after the cells reached about 60% confluence for 48 h. Then, the following experiments were carried out.

### 2.3. ALF Rat Model and Cell Administration

The rat ALF model was established by a single intraperitoneal injection of carbon tetrachloride (CCl_4_). For the survival rate observation experiment (*n* = 10/group), the rats received 50% (*v*/*v*) CCl_4_ vegetable oil solution at the dosage of 3 mL/kg body weight, while the dosage in other experiments was 2.5 mL/kg body weight. Rats received the same volume of vegetable oil were taken as the control group. 6 h after the administration of CCl_4_, the ALF rats were randomly divided into three groups (*n* = 8/group): normal saline (NS) group, the rats received 5 mL/kg body weight of NS; MSC group, the rats received 1 × 10^7^ cells/kg body weight of MSCs; and Pre-MSC group, the rats received 1 × 10^7^ cells/kg body weight of Pre-MSCs. The treatments were performed via a tail vein, and the cell suspension concentration was 2 × 10^6^ cells/mL. To evaluate the outcomes of ALF animals, the survival of rats after treatment was recorded each day at the same time till the sixth day. Besides, in the functional experiments, the blood was collected through the angular vein at 24 h and 48 h after treatment. Finally, the rats were sacrificed by anesthesia at 48 h after treatment to collect liver tissues which were then fixed in formalin solution for 24 h.

### 2.4. Liver Function and Histological Evaluation

Serum alanine aminotransferase (ALT), aspartate aminotransferase (AST), total bilirubin (TBIL), and albumin (ALB) were detected by a chemistry analyzer (VITROS 5600, Ortho, Rochester, USA). The formalin-fixed and paraffin-embedded liver tissues were cut into 4 *μ*m slices for hematoxylin and eosin (HE) staining and immunohistochemical analyses. HE staining was observed with a microscope (Nikon TE2000U, Tokyo, Japan), and 5 microimages with 100x magnification were taken randomly to analyze the necrotic ratio of the liver by Image J.

### 2.5. In Situ Apoptosis Detection and Immunohistochemical Staining

In situ apoptosis detection was carried out by TUNEL assay using a commercial kit following the manufacturer's instructions (Roche, Mannheim, Germany). Briefly, the slices of liver tissues were treated with deparaffinage, rehydration, protease K incubation, equilibration buffer incubation, TUNEL reaction mixture incubation, and seal. For immunohistochemical staining, the antigen repair method was employed with anti-rat Ki-67 (Abcam, Cambridge, UK) as the primary antibody. Briefly, deparaffinage, rehydration, antigen repair, inactivation of endogenous peroxidase, block, anti-rat Ki-67 antibody (1 : 200) and secondary antibody (1 : 1000) incubation, and seal were performed step by step. At last, the slices were observed using the microscope, and 5 microimages with 200x magnification were taken randomly to analyze the positive cells.

### 2.6. Cell Labeling and Homing Experiments In Vivo

MSCs and Pre-MSCs were labeled by CM-Dil (Invitrogen, Carlsbad, USA) according to the manufacturer's instructions. In brief, after digestion with trypsin and centrifugation, the cells were resuspended with PBS containing 3 *μ*M CM-Dil and then incubated for 5 min at 37°C, then at 4°C for 15 min. The medium was discarded, and the cells were washed 3 times with PBS. Finally, the NS cell suspension was prepared with a concentration of 2 × 10^6^ cells/mL. A small part of the CM-Dil-labeled cells was cultured in DMEM/F-12 culture medium. When the growth was stable, the labeling rate was analyzed by comparing images of fluorescent and white light, using a fluorescence microscope. After 6 h of CCl_4_ administration at 2.5 mL/kg body weight, the ALF rats were injected with MSCs or Pre-MSCs (1 × 10^7^ cells/kg body weight) via a tail vein. The rats injected the same volume of NS were used as control. After 24 h, the livers were collected and frozen at -80°C. The frozen 4 *μ*m slices were prepared, and the distribution of labeled cells in liver tissues was observed with a fluorescence microscope. Microimages were taken randomly with 200x magnification to analyze the relative optical density of each group.

### 2.7. Western Blot and Surface CXCR4 Expression Detection

The protein expression levels of CXCR4 and c-Met in MSCs and Pre-MSCs were detected by Western blot. According to the instructions of the BCA kit (Beyotime Biotechnology, Shanghai, China), the total cell proteins were collected. Anti-rat CXCR4 (1 : 1000, Diagbio, Hangzhou, China), c-Met (1 : 2000, Diagbio, Hangzhou, China), tubulin (1 : 1000, Cell Signaling Technology, Danvers, MA, USA), and the corresponding secondary antibodies were applied. The gray values were analyzed by Image J. The surface CXCR4 expression was detected by flow cytometry. Briefly, P4 MSCs or Pre-MSCs reaching ∼90% confluence were digested and washed with PBS containing 0.1% bull serum albumin and then stained with PE Anti-Human CD184/CXCR4 Antibody (Elabscience, Wuhan, China). After another washing, surface CXCR4 expression was determined by a FACS Calibur flow cytometer (BD Biosciences, San Jose, CA, USA).

### 2.8. Transwell Migration Assay

Transwell chambers (Corning, New York, USA) with a 6.5 mm diameter and a polyethylene terephthalate (PET) track-etched membrane with 8.0 *μ*m pores were used in the Transwell migration assay. 1 × 10^4^ cells were seeded in the upper chambers in 200 *μ*L of DMEM/F-12 medium containing 0.1% (g/mL) BSA, and 600 *μ*L DMEM/F-12 culture medium supplemented with 10% (*v*/*v*) FBS was added in the lower chambers. For the chemotaxis assay, SDF-1 (PeproTech, Rocky Hill, NJ, USA) at concentrations of 0, 10, 20, 50, and 100 ng/mL were used as the lower chamber medium. In the chemotaxis inhibition assay, MSCs and Pre-MSCs were incubated with 10 *μ*g/mL AMD3100 (MedChemExpress, Shanghai, China), an antagonist of CXCR4 for 2 h before seeded in the upper chambers. The cells without preincubation were used as control. The concentration of SDF-1 in the lower chambers was 100 ng/mL. After 24 h culture, cells in the upper chambers were removed and the membranes were fixed with 4% paraformaldehyde for 20 min. Then, the cells migrated to the lower side of the filter were stained with 0.5% crystal violet for 10 min. At last, the cells were observed with the microscope, and 5 microimages with 100x magnification were taken randomly to analyze the migrated cells.

### 2.9. Statistical Analysis

The data were exhibited as mean ± standard deviation (±SD) and analyzed by SPSS19.0 software (V19.0 for Windows; SPSS, Inc., Chicago, IL, USA). Statistical significance was determined via a one-way analysis of the variance (ANOVA), and Student's *t*-test was used when there were two groups. Kaplan-Meier survival plots were generated with survival curves, and the differences were compared by the log-rank test. *P* < 0.05 was considered statistically significant, and *P* < 0.01 and *P* < 0.001 were considered highly significant.

## 3. Results

### 3.1. Characterizations of MSCs

**Figure 1:**
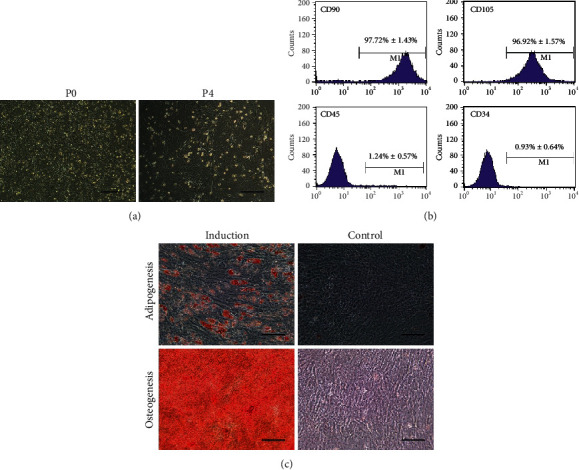
Characterizations of MSCs. (a) Morphologies of primary and subcultured (P4) MSCs. (b) Flow cytometry analysis of the immunophenotypes of P4 MSCs. MSCs were positive for CD90 and CD105, but negative for CD45 and CD34 (*n* = 3). (c) Differentiation potentials of MSCs. Adipogenesis and osteogenesis of MSCs were confirmed by Oil Red O staining and Alizarin Red S staining after culturing in corresponding induction or control medium. Bar = 100 *μ*m.

The whole bone marrow adherence method was employed to isolate and culture MSCs from rats. In primary culture, the short spindle cells could be seen to grow adhering to the wall following the removal of blood cells with medium changes (Figure 1(a) P0). During subculture, the volume of the cell increased, showing long spindle and polygonal growth shape (Figure 1(a) P4). The immunophenotypes of P4 cells were detected by flow cytometry. The results showed that the cells exhibited high expression of CD90 and CD105, which were surface markers of mesenchymal stem cells, but the cells were negative for hematopoietic cell markers, such as the CD45 and CD34 (Figure 1(b)). Furthermore, the multidifferentiation potential of the cultured cells was confirmed by inducing lipogenesis and osteogenesis. As shown in Figure 1(c), lipid droplets which were positively stained by Oil Red O were observed in cells cultured in adipogenic medium, while the cells induced by osteogenic medium were positive for Alizarin Red S staining, indicating the formation of calcium salt. There was no staining in the corresponding control groups. All these results confirmed the successful isolation of the MSCs.

### 3.2. The Effect of IL-1*β*-Pretreated MSCs on Survival Rate and Liver Function

**Figure 2:**
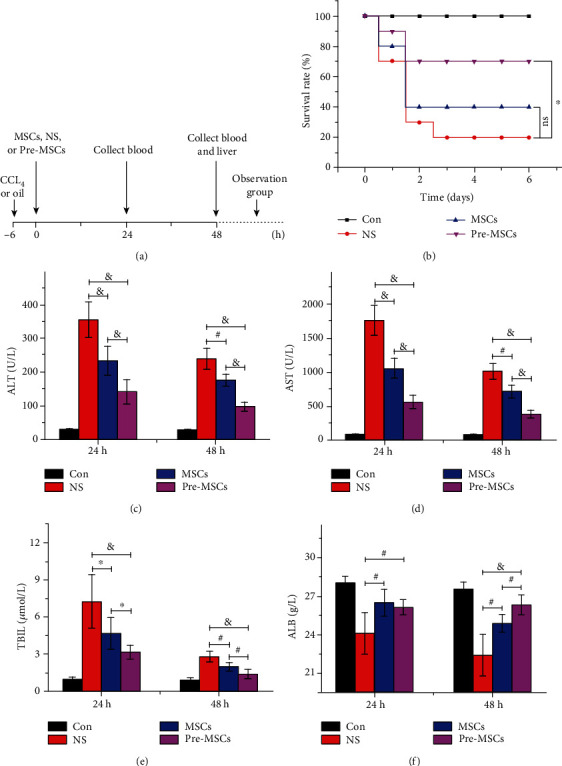
IL-1*β* pretreatment improved the protective effect of MSCs on the survival rate and liver function in ALF rats. (a) Flow chart of *in vivo* experiments. The rats were intraperitoneally administrated with CCL_4_ to induce ALF or with oil as control, followed by intravenous injection with NS, MSCs, or Pre-MSCs 6 h later. The observation group was observed for six days, and the specimens of rats in the other groups were collected at 24 h and 48 h after treatment. (b) The survival rate of rats dealt with different protocol was recorded every day (*n* = 10). (c) The levels of serum ALT (c), AST (d), TBIL (e), and ALB (f) were detected (*n* = 8). ^∗^*P* < 0.05, ^#^*P* < 0.01, and ^&^*P* < 0.001.

In order to evaluate the impact of IL-1*β* pretreatment on the efficacy of MSCs in ALF, a rat ALF model was established by a single intraperitoneal injection of CCl_4_, and the rats were divided into four groups randomly: control group, NS group, MSC group, and pre-MSC group (Figure 2(a)). No rat died in the control group till the end of the observation. The survival rate of the MSC group was 40% on the sixth day, which was higher than 20% of the NS group, but the overall survival difference was not statistically significant. Meaningfully, IL-1*β* pretreatment further increased the survival rate of MSC treatment to 70%, and the overall survival of the Pre-MSC group was significantly higher than that of the NS group (*P* < 0.05, Figure 2(b)).

The serum was collected at 24 h and 48 h after treatment, and the ALF, AST, TBIL, and ALB were detected to evaluate the changes of liver function. As shown in Figures 2(c)–2(f), the MSC group had significantly better liver function compared to the NS group, while IL-1*β* pretreatment further notably improved the efficacy of MSCs. At 24 h, ALT, AST, and TBIL in the Pre-MSC group were 141.71 ± 34.98 U/L, 556.14 ± 97.32 U/L, and 3.14 ± 0.54 *μ*mol/L, respectively, which were significantly lower than the corresponding 233.43 ± 2.30 U/L, 1049.86 ± 150.35 U/L, and 4.67 ± 1.28 *μ*mol/L in the MSC group (*P* < 0.05). For ALB, an indicator of disease consumption and liver synthesis function, it was 26.11 ± 0.58 g/L in the Pre-MSC group and 26.48 ± 1.03 g/L in the MSC group, which were both significantly higher than that in the NS group at 24.1 ± 1.61 g/L (*P* < 0.01), but there was no significant difference between the two MSC therapy protocols. At 48 h, the ALT, AST, TBIL, and ALB in the Pre-MSC group were 98.29 ± 12.24 U/L, 376.14 ± 60.09 U/L, 1.37 ± 0.37 *μ*mol/L, and 26.31 ± 0.76 g/L, respectively, which were significantly better than those in the MSC group (*i.e.*, ALT 176.67 ± 17.61 U/L, AST 713.83 ± 91.52 U/L, TBIL 1.9 ± 0.32 *μ*mol/L, and ALB 24.88 ± 0.69 g/L, *P* < 0.01). Taken together, these results strongly indicated that the administration of MSCs pretreated with IL-1*β* notably improved the liver function in ALF compared with normal MSC injection.

### 3.3. The Effect of IL-1*β*-Pretreated MSCs on Liver Necrosis, Hepatocyte Apoptosis, and Proliferation

**Figure 3:**
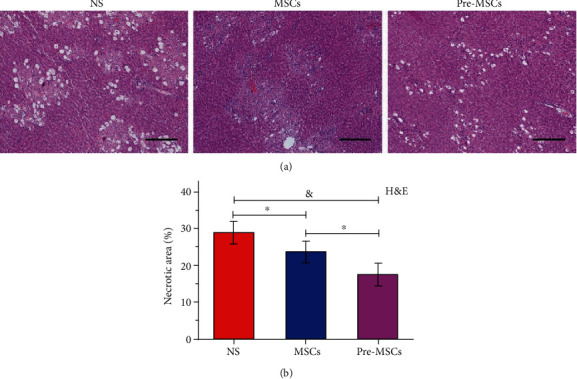
The improvement effect of IL-1*β* pretreatment on MSCs for liver necrosis. (a) Images from HE staining of livers at 48 h after NS, MSC, or Pre-MSC administration. (b) Relative necrotic areas of liver specimens from HE staining (*n* = 5). Bar = 200 *μ*m. ^∗^*P* < 0.05 and ^&^*P* < 0.001.

The HE staining of liver tissues collected after 48 hours of treatment was carried out to assess the impact of IL-1*β* pretreatment on MSC efficacy histologically. As shown in Figure 3(a), in the NS group, there were large areas of necrotic liver tissues which were obviously ameliorated after MSC or Pre-MSC administration, and the Pre-MSC group exhibited the mildest necrosis. Further statistical analysis showed that the relative necrosis area of the MSC group was 23.72% ± 2.88%, which was significantly lower than that of the NS group (28.95% ± 3.00%). IL-1*β* pretreatment further improved the efficacy of MSCs with a relative necrosis area of 17.64% ± 3.07%, which was significantly lower than the other two groups (*P* < 0.01, Figure 3(b)).

**Figure 4:**
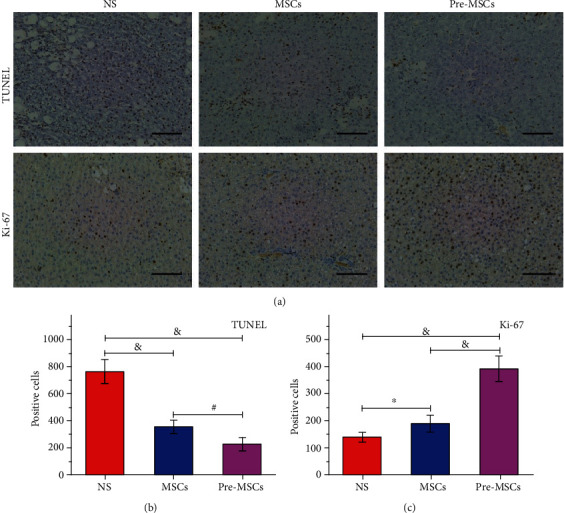
The improvement effect of IL-1*β* pretreatment on MSCs for hepatocyte apoptosis and proliferation treatment. (a) Images from TUNEL assays and immunohistochemical staining of Ki-67 of livers at 48 h after NS, MSC, or Pre-MSC administration. (b, c) Statistical analysis of TUNEL-positive and Ki-67-positive hepatocytes of livers at each group (*n* = 5). Bar = 100 *μ*m. ^∗^*P* < 0.05, ^#^*P* < 0.01, and ^&^*P* < 0.001.

In order to clarify the effect of MSCs and Pre-MSCs on hepatocyte apoptosis and liver reconstruction, the TUNEL staining and immunohistochemical staining of Ki-67 were performed. The results of TUNEL staining showed that the rats in the NS group had the most serious hepatocyte apoptosis, and the number of positive cells was 765.00 ± 86.97. Both kinds of MSCs significantly improved the condition in the NS group (*P* < 0.001), while the Pre-MSCs had a better efficacy (apoptotic hepatocytes: Pre-MSCs 225.4 ± 51.03*vs.* MSCs 355.4 ± 46.01, *P* < 0.01, Figures 4(a) and 4(b)). The trend of Ki-67 immunohistochemical staining was opposite to that of TUNEL. As shown in Figures 4(a) and 4(c), the number of proliferating cells in the NS, MSC, and Pre-MSC groups was 139.4 ± 17.18, 189.2 ± 29.99, and 391.8 ± 46.58, respectively, and the Pre-MSCs had the strongest effect in promoting hepatocyte proliferation (*P* < 0.05). Overall, these findings *in vitro* supported the improved effect of IL-1*β*-pretreated MSCs on liver necrosis, cell apoptosis, and proliferation compared with MSCs.

### 3.4. Enhanced Homing Ability of Pre-MSCs to the Liver of ALF Rats

**Figure 5:**
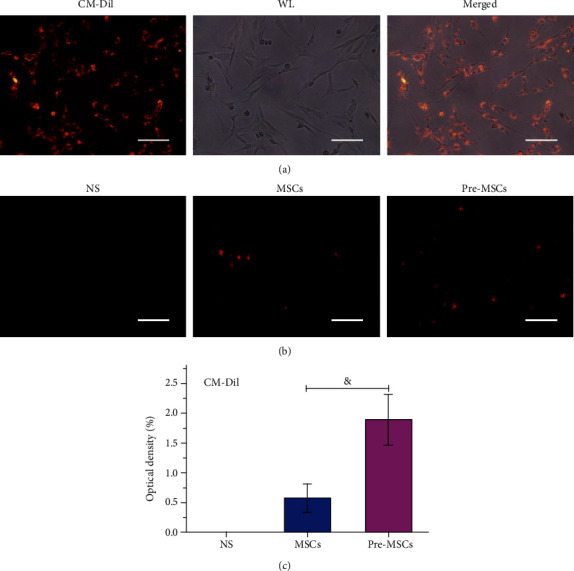
IL-1*β* pretreatment enhanced MSCs homing to livers in ALF rats. (a) Fluorescence and white light (WL) images of CM-Dil-labeled MSCs. Bar = 100 *μ*m. (b) Fluorescence images of livers at 24 h after NS, MSC, or Pre-MSC administration. (c) Relative optical densities of livers at each group (*n* = 5). Bar = 200 *μ*m. ^&^*P* < 0.001.

To investigate the homing ability of MSCs and Pre-MSCs, CM-Dil-labeled cells were used. As shown in Figure 5(a), almost all the cells could be labeled positively, and the labeling had no obvious effect on the cell morphology. The livers were collected at 24 h after cell administration. The frozen sections were observed by a fluorescence microscope, and the optical density was analyzed by Image J. As shown in Figures 5(b) and 5(c), there was no red fluorescence in the control group. The Pre-MSC group had the most obvious fluorescence, with a relative fluorescence density of 1.88% ± 0.42%, which was significantly higher than that in the MSC group (*i.e.*, 0.57% ± 0.23%, *P* < 0.001).

### 3.5. Enhanced CXCR4 Expression of IL-1*β*-Pretreated MSCs

**Figure 6:**
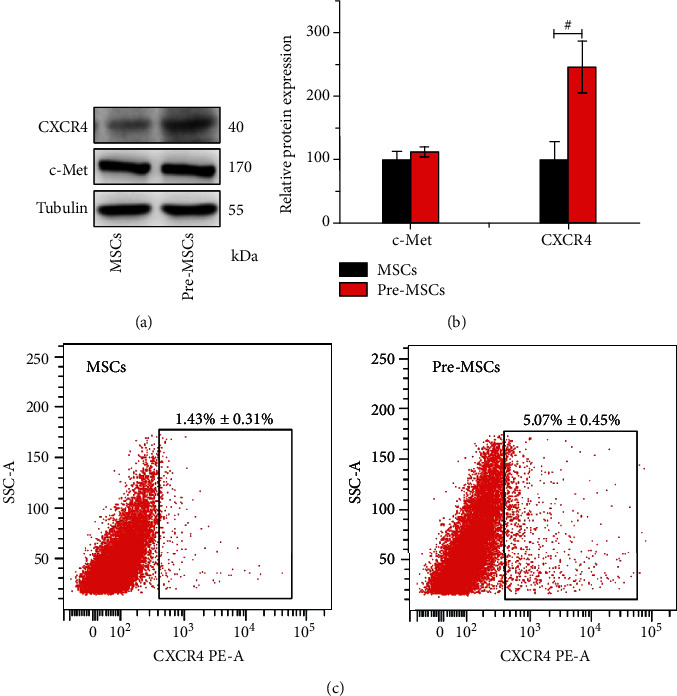
IL-1*β* pretreatment enhanced CXCR4 expression of MSCs. (a) Western blot analysis of CXCR4 and c-Met protein levels of Pre-MSCs and MSCs. (b) Statistical analysis of relative protein expression to tubulin (*n* = 3). (c) Flow cytometry analysis of CXCR4 surface expression (*n* = 3).

CXCR4 and c-Met are important cell surface receptors determining the migration ability of MSCs [9]. In order to preliminarily explore the mechanism of the enhanced homing ability of IL-1*β*-pretreated MSCs to the damaged liver, the expressions of these two receptors were detected by Western blot. As shown in Figures 6(a) and 6(b), IL-1*β* pretreatment significantly upregulated the expression of CXCR4 of MSCs, and the gray value of CXCR4 in Pre-MSCs was about 2.46 times of that in MSCs (*P* < 0.001). However, there was no significant difference in c-Met expression between the two kinds of MSCs. For the surface expression which is important for the function of chemokine receptor, flow cytometry was carried out to detect the CXCR4 in MSC membrane. As shown in Figure 6(c), the positive rate of CXCR4 in the Pre-MSC group was 5.07% ± 0.45%, which was significantly higher than 1.43% ± 0.31% in the MSC group (*P* < 0.001).

### 3.6. Enhanced Migration Ability of Pre-MSCs to SDF-1 Based on CXCR4

**Figure 7:**
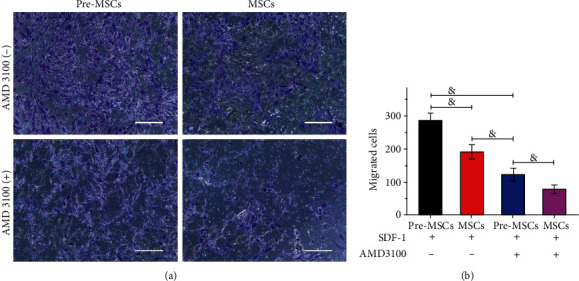
IL-1*β* pretreatment enhanced migration ability of MSCs to SDF-1 based on CXCR4. (a) Images from Transwell migration assay of Pre-MSCs and MSCs with or without AMD3100 preincubation for 2 h. (b) Number of migrated cells of different groups (*n* = 5). ^&^*P* < 0.001.

SDF-1 is an important ligand of CXCR4. In order to further explore the impact of the IL-1*β* pretreatment-induced enhancement of CXCR4 expression on the migration ability of MSCs, the Transwell migration assay was carried out with SDF-1 and AMD3100 application. The results revealed that MSCs exhibited a dose-dependent chemotaxis to SDF-1, and the maximum migration was achieved at the concentration of 100 ng/mL (Figure S1), which was then used in the chemotaxis inhibition assay. As shown in Figure 7, the number of migrated cells in the Pre-MSC group was significantly higher than that in the MSC group (285.6 ± 23.02*vs.*191 ± 21.39, *P* < 0.001). The application of AMD3100 significantly reversed the improving effect of IL-1*β* pretreatment on MSC migration (*P* < 0.001), and the number of migrated cells in this group was only 123.2 ± 18.67, which, however, was still higher than 78.2 ± 12.62 of the MSC+AMD3100 group (*P* < 0.001).

## 4. Discussion

Since ALF is fast-progressing and lethal, there is no effective treatment other than liver transplantation [1]. With powerful metabolic and functional supporting abilities for inflammatory diseases, MSCs may be an effective therapeutic strategy for ALF, or at least provide a better chance for patients waiting for a liver donor. MSCs have many other advantages, such as low immunogenicity, easy access, and no ethical issues, making it serve as a replacement of hepatocytes in ALF therapy [19]. Numerous preclinical experiments have confirmed that the application of MSCs can effectively reduce mortality and improve the liver function and pathophysiological process of ALF [6, 8]. Although MSCs have wide sources, the most common source is bone marrow, and MSCs isolated from bone marrow have been studied relatively thoroughly [20]. Therefore, bone marrow MSCs were chosen in this study. The whole bone marrow adherence, the simplest method, has minor damage to cells and a lower cell loss rate than other protocols. Therefore, it was employed to isolate and culture MSCs from rats in this study. As shown in the characterization experiments of cultured cells, the expression rates of CD90 and CD105 were greater than 95%, while those of CD45 and CD34 were less than 2%, and the good multidifferentiation potential was also verified.

The efficacy of MSCs on ALF could be significantly improved by effective modification or pretreatment [10, 21]. Although MSCs can differentiate into hepatocytes, recent studies have shown that microenvironment created by MSCs is the critical factor for their efficacy in improving the pathophysiological process of liver diseases [8]. Meanwhile, the modeling of inflammatory environment on MSCs plays an important role. Several studies have shown that MSCs could promote the progress of inflammation in the early stage of the disease [22]. As a major inflammatory factor, it is unclear whether IL-1*β* pretreatment will affect the efficacy of MSCs on ALF. As reported previously, 20 ng/mL IL-1*β* could more significantly improve the paracrine signaling and efficacy than 10 ng/mL without changing the characteristics of MSCs [23, 24]. Hence, 20 ng/mL was chosen as the appropriate stimulation concentration in this study. The results of this study showed that the survival rate and liver function were significantly improved by the MSCs administration, while IL-1*β* pretreatment significantly enhanced the efficacy of MSCs. Compared with the MSC and NS groups, the rats received Pre-MSCs had the smallest area of liver necrosis, the smallest number of apoptotic hepatocyte, and the largest number of proliferating cells. All of these results indicated that Pre-MSCs could better improve the pathophysiological process of ALF than MSCs.

When creating an extracellular microenvironment that could improve the pathophysiology of the disease, MSCs may play an initiating role by cell-cell contact and produce a small amount of paracrine cytokines, such as prostaglandin E2 and TNF-*α*-stimulated gene 6 protein (TSG-6). Actually, the nonparenchymal cells, such as macrophage and mesothelial cells, are the effector cells that can create a beneficial environment [25, 26]. Shi et al. applied human MSCs to treat a pig model of fulminant hepatic failure. By observing the liver based on human-specific primers, they found that the most significantly changed cytokines in the serum were from the model animal itself rather than human MSCs. Further analysis of gene expression of MSCs, the treatment of animals, and the control animals revealed that the implanted MSCs altered cytokine responses of damage through paracrine effects [27]. Therefore, the paracrine effects play a vital role in the effect of MSCs, and by improving the homing ability of MSCs to the damaged sites, the paracrine enhancement may be an important strategy to improve the therapeutic ability of MSCs.

Sun et al. confirmed that IL-1*β* could significantly enhance the migration ability of squamous cell carcinoma cells [17], while Kao et al. declared that IL-1*β* could induce COX-2 expression and promote the invasion of endometrial MSCs [28]. However, the impact of IL-1*β* pretreatment on the homing ability of MSCs, especially the bone marrow-derived MCSs, to the damaged liver is still unclear. The results of this study showed that IL-1*β* pretreatment significantly enhanced the distribution of MSCs in the damaged liver, indicating that IL-1*β* pretreatment improved the efficacy of MSCs at least partially by enhancing the homing of MSCs.

A variety of molecules can affect the migration and homing ability of MSCs. Due to the high levels of HGF and SDF-1 in the injured liver, HGF-c-Met and SDF-1-CXCR4 axis are common signals for the homing ability of MSCs to the liver [29, 30]. Zhang et al. showed that P5 MSCs, which had a higher level of c-Met, exhibited stronger migration ability to the injured liver and better efficacy than P10 cells [29]. As a specific receptor of SDF-1, which is one of the most powerful chemokines, CXCR4 has been widely studied in the homing of various cells, including that of MSCs to the injured liver. Most of these studies confirmed the critical role of CXCR4 [21]. The Western blot results of this study showed that the expression of CXCR4 in Pre-MSCs was significantly increased compared to cells without IL-1*β* pretreatment, but the quantity of c-Met expression was constant. Further flow cytometry detection showed that the cell surface CXCR4 expression was also increased after IL-1*β* pretreatment. In the migration experiments *in vitro*, the Pre-MSCs exhibited significantly improved migration ability towards SDF-1 compared to MSCs, which was reversed by CXCR4 antagonist, but still stronger than MSCs in AMD3100 condition. These results indicated that the enhanced CXCR4 expression was at least partially responsible for the increased MSC homing ability induced by IL-1*β* pretreatment.

## 5. Conclusions

To sum up, MSCs treatment could significantly improve the survival rate, liver function, liver necrosis, and hepatocyte apoptosis of ALF rats, and liver reconstruction was also enhanced. More meaningfully, IL-1*β* pretreatment significantly improved the above-mentioned efficacy of MSCs. In addition, we confirmed that IL-1*β* pretreatment significantly enhanced the homing ability of MSCs to the damaged liver, which was at least partially benefited from the promotion effect of IL-1*β* on CXCR4 expression. Therefore, by increasing the expression of CXCR4, IL-1*β* pretreatment could enhance the homing ability of MSCs and further improve the efficacy of MSCs on ALF.

## Data Availability

The data used to support the findings of this study are available in the study findings, and more details are available from the corresponding authors upon request.

## Supplementary Materials

Supplementary Materials Figure S1: dose-dependent chemotaxis to SDF-1 of MSCs. Images from Transwell migration assay of MSCs to SDF-1 of different concentrations, and numbers of migrated cells of different groups (*n* = 5). ^∗^*P* < 0.05 and ^∗^*P* < 0.001.
