# Supplementary material for: IL-1β Pretreatment Improves the Efficacy of Mesenchymal Stem Cells on Acute Liver Failure by Enhancing CXCR4 Expression
Source: Stem Cells Int. 2020 Jul 7;2020:1498315. doi: 10.1155/2020/1498315 (PMC7364198; doi:10.1155/2020/1498315)
Supplement: Supplementary Materials — Figure S1: dose-dependent chemotaxis to SDF-1 of MSCs. Images from Transwell migration assay of MSCs to SDF-1 of different concentrations, and numbers of migrated cells of different groups (n = 5). ∗P < 0.05 and ∗P < 0.001. [file 1498315.f1.docx]

**Supplementary figure**


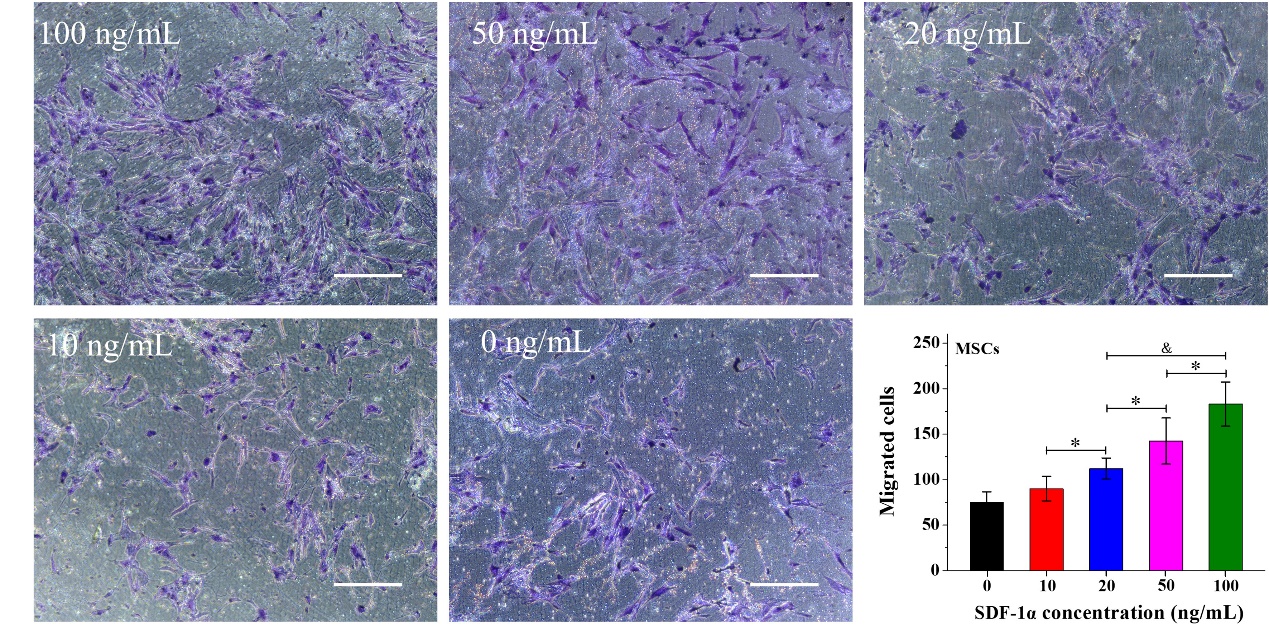


**Figure S1.** Dose-dependent chemotaxis to SDF-1 of MSCs. Images from Transwell migration assay of MSCs to SDF-1 of different concentrations, and numbers of migrated cells of different groups (n = 5). ^∗^*P <* 0 05, &P < 0 001.
